# Detection of antibodies to the α4β7 integrin binding site on HIV-1 gp120 V2 loop using a novel cell adhesion assay

**DOI:** 10.1186/1742-4690-9-S2-P71

**Published:** 2012-09-13

**Authors:** M Rao, N Karasavvas, A Pinter, H Liao, M Bonsignori, B Mathieson, S Zolla-Pazner, BF Haynes, NL Michael, JH Kim, CR Alving, KK Peachman

**Affiliations:** 1 U.S. Military HIV Research Program, WRAIR, Silver Spring, MD, USA; 2 Armed Forces Research Institute of Medical Science (AFRIMS), Bangkok, Thailand; 3 The Public Health Research Institute Center at the International Cente, Newark, NJ, USA; 4 Duke Human Vaccine Institute Duke University Medical Center, Durham, NC, USA; 5 Office of AIDS Research, National Institutes of Health, Bethesda, MD, USA; 6 NYU School of Medicine, New York, NY, USA; 7 United States Military HIV Research Program, WRAIR / HJF, Silver Spring, MD, USA

## Background

The gut mucosal homing integrin receptor α4β7 present on activated CD4+ T-cells interacts with the HIV-1 gp120 second variable loop (V2). Case control analysis of the RV144 phase III vaccine trial showed that antibodies induced by the vaccine bound to a MuLV-gp70 scaffolded V1V2 loop of gp120 (V1V2-gp70) and correlated inversely with infection. These, and other data, generate the hypothesis that the vaccine-elicited antibodies may have been involved in limiting HIV-1 acquisition. We have developed a high-throughput assay to evaluate antibodies that block α4β7 binding. We have named this the RAP assay.

## Methods

Plates were coated with either MAdCAM-1, the natural ligand of α4β7, or streptavidin followed by the addition of biotinylated cyclic-V2 peptides (strain 92TH023 or MN). Plasma and purified IgG antibodies from RV144 volunteers, conformational mAbs specific for V2 (697), for V2 and V3 (PG9, PG16), or for V2 linear epitopes (CH58 and CH59, cloned from RV144 vaccinee B cells) were then added to the peptide-coated plates followed by RPMI8866 cells, which constitutively express α4β7. Cell binding/inhibition was assessed by AlamarBlue. Anti-α4β7-specific mAb (ACT-1) served as a control. In separate experiments, plasma, IgG, or mAbs were tested in a competition assay using V1V2-gp70.

## Results

ACT-1 inhibited the binding of both MAdCAM-1 and cyclic-V2 peptides to α4β7 by 65-85%, while CH58 and CH59 inhibited the α4β7-cyclic-V2 peptide binding by 37-45% in a dose-dependent manner. PG9, PG16, and 697 did not inhibit the binding of V2-peptides to the cells. However, in the competition assay, 697 and PG9 mAbs inhibited V1V2-gp70 binding to α4β7. Some of the RV144 plasma and IgG inhibited binding to 92TH023 or MN-V2-peptides.

## Conclusion

We have developed a novel high-throughput reproducible assay for assessing α4β7-specific blocking antibodies. The above results raise the hypothesis that anti-V2 loop antibodies may play a role in regulation of gp120-α4β7 interaction.

